# Obstetric Outcomes Among Underage Mothers: Age-Stratified Analysis from a Romanian Hospital-Based Cohort

**DOI:** 10.3390/medicina62061032

**Published:** 2026-05-26

**Authors:** Florin Mihai Sandor, Izabella Petre, Ion Petre, Florina Buleu, Cris Virgiliu Precup, Roxana Furau, Tiberiu Buleu, Maria Ioana Ardelean, Adrian Loichita, Cristian George Furau

**Affiliations:** 1 Multidisciplinary Doctoral School, “Vasile Goldis” Western University of Arad, 310414 Arad, Romania; sandor.florin@uvvg.ro (F.M.S.); furau.cristian@uvvg.ro (C.G.F.); 2 Department of Life Sciences, “Vasile Goldis” Western University of Arad, 310414 Arad, Romania; precup.cirs@uvvg.ro; 3 Department of Obstetrics and Gynecology, “Victor Babes” University of Medicine and Pharmacy, Murgu Sq. No. 2, 300041 Timisoara, Romania; petre.izabella@umft.ro; 4 Department of Functional Sciences, Medical Informatics and Biostatistics Discipline, ”Victor Babeş” University of Medicine and Pharmacy, Eftimie Murgu Sq. No. 2, 300041 Timisoara, Romania; 5 Department of Cardiology, “Victor Babes” University of Medicine and Pharmacy, Eftimie Murgu Sq. No. 2, 300041 Timisoara, Romania; 6 Department of General Medicine, Faculty of Medicine, “Vasile Goldis” Western University of Arad, 310414 Arad, Romania; furau.roxana@uvvg.ro; 7 Faculty of General Medicine, “Victor Babes” University of Medicine and Pharmacy, 300041 Timisoara, Romania; tiberiu.buleu@umft.ro; 8 Doctoral School, Faculty of General Medicine, “Victor Babes” University of Medicine and Pharmacy, 300041 Timisoara, Romania; ioana.mosutiu@umft.ro (M.I.A.); adrian.loichita@umft.ro (A.L.)

**Keywords:** adolescent pregnancy, obstetric outcomes, underage mothers, Romania, cesarean delivery, maternal morbidity

## Abstract

*Background and Objectives*: Adolescent pregnancy is traditionally associated with increased obstetric risk, particularly among very young adolescents. Romania continues to report one of the highest adolescent birth rates in the European Union, yet age-stratified clinical data on obstetric outcomes among minors remain limited. This study aimed to evaluate maternal and intrapartum outcomes among underage mothers (<18 years), stratified by age (<15, 15–16, and 17 years), and to identify predictors of adverse obstetric outcomes. *Materials and Methods*: We conducted a retrospective cohort study of all live births and stillbirths among mothers aged 12–17 years recorded between 2020 and 2024 at a secondary maternity hospital in Romania. Data were extracted from standardized obstetric and neonatal records. Primary outcomes included preterm birth (<37 weeks), cesarean delivery, and intrapartum complications. Group comparisons were performed using non-parametric tests and the χ^2^ test. Multivariable logistic regression models were used to assess independent associations between maternal age and obstetric outcomes. *Results*: The cohort comprised 763 adolescent mothers aged 12–17 years. No significant differences were observed across age groups in gestational age at birth, preterm birth rate, fetal presentation, or mode of delivery. In multivariable analyses, younger maternal age was not independently associated with preterm birth (adjusted odds ratios [aORs] 0.87–1.21) or cesarean delivery (aORs 0.74–1.08). Obstetric factors, such as non-cephalic presentation and characteristics of membrane rupture, were the main predictors of adverse outcomes. Temporal analyses demonstrated stable outcome patterns across the study period, with no significant interaction between maternal age and year of delivery. *Conclusions*: In this tertiary-care cohort, very young maternal age (<15 years) was not associated with poorer obstetric outcomes compared with older adolescents. These findings suggest that standardized antenatal surveillance and intrapartum management may mitigate age-related obstetric risk among underage mothers. Further population-based studies are warranted to contextualize these results within broader healthcare access and social determinants of adolescent pregnancy. Cesarean section rates were high across all groups (~50%), with no significant differences by maternal age.

## 1. Introduction

Adolescent pregnancy remains a major global public health concern, with substantial consequences for maternal and neonatal outcomes worldwide. It is estimated that approximately 12 million girls aged 15–19 years give birth each year, alongside nearly 777,000 births among girls younger than 15 years, reflecting a persistent burden of early childbearing across diverse settings [1,2]. According to the World Health Organization (WHO), complications related to pregnancy and childbirth are among the leading causes of mortality in adolescent girls aged 10–19 years, including in middle- and high-income countries, underscoring the clinical relevance of adolescent maternal health beyond low-resource regions [1,3]. Although global adolescent fertility rates have declined over the past two decades, progress has been uneven, with pronounced geographic disparities shaped by socioeconomic inequalities, access to reproductive health services, and prevailing cultural norms [2,4,5].

A substantial body of epidemiological and clinical research has documented increased obstetric and neonatal risks associated with adolescent pregnancy, particularly among very young adolescents. Meta-analyses and large population-based studies have reported higher rates of preterm birth, low birth weight, obstructed labor, maternal morbidity, and perineal trauma among younger adolescents compared with adult women [5,6,7].

Biological immaturity—manifested through incomplete pelvic growth, reduced uterine and placental perfusion, and increased vulnerability to nutritional deficiencies—has been proposed as a contributing mechanism to adverse outcomes, although its relative impact appears to vary depending on healthcare access and contextual factors [5,7,8]. Multicountry analyses coordinated by the WHO further suggest that girls aged ≤14 years face a disproportionately elevated risk of severe maternal and perinatal complications compared with older adolescents, despite being consistently underrepresented in obstetric research [1,5,7,8].

Within Europe, adolescent pregnancy is relatively uncommon; however, Romania consistently reports the highest adolescent birth rates among European Union (EU) member states. Data from the Euro-Peristat Project indicate that, in 2022, Romania recorded a birth rate of approximately 33 per 1000 girls aged 15–19 years—more than three times the EU average of roughly 10 per 1000 [9,10]. Romania is also one of the few EU countries reporting a sustained number of births among girls aged 10–14 years, with national statistics documenting over 700 such births annually over the past decade [10]. According to data from the National Institute of Statistics (INS), approximately 16,000 births occur each year among minors (<18 years), including 10,000–11,000 births to 17-year-olds, 5000–6000 births to adolescents aged 15–16 years, and 500–700 births annually among girls younger than 15 years, highlighting a distinct and persistent epidemiological profile within the EU [11,12,13].

These patterns reflect not only demographic trends but also underlying social determinants, including gaps in comprehensive sexual education, limited access to contraception, early school dropout, and marked socioeconomic inequalities affecting rural and marginalized communities in Romania [14,15]. Together, these factors contribute to both the high incidence of adolescent pregnancy and the complexity of associated health outcomes.

Despite the magnitude of this public health issue, detailed clinical data describing obstetric outcomes among Romanian minors remain limited. National surveillance systems primarily rely on aggregated demographic indicators and lack granular obstetric variables such as fetal presentation, duration of membrane rupture, intrapartum complications, or perineal trauma, constraining the clinical interpretability of available data [13]. Moreover, adolescent pregnancies are frequently analyzed as a single age category (<20 years), potentially obscuring important heterogeneity in risk profiles across adolescent subgroups, particularly among very young adolescents (<15 years), who may represent a distinct clinical population [5,8]. An additional unresolved issue concerns the relative contribution of biological age versus contextual factors to adverse obstetric outcomes in adolescent pregnancies. Evidence from high-resource settings suggests that, after adjustment for socioeconomic status, antenatal care utilization, and nutritional factors, obstetric risks among adolescents may decrease substantially and, in some cases, approximate those observed in adult women [5,8,16].

These findings raise critical questions regarding the extent to which biological immaturity alone accounts for adverse outcomes, as opposed to modifiable social and healthcare-related determinants. In Romania, where adolescent pregnancies disproportionately affect socioeconomically vulnerable populations, disentangling these influences is essential for informing effective prevention strategies and clinical care models.

To address these gaps, the present retrospective study analyzed all births among minors (<18 years) delivered at a secondary obstetric center in Western Romania over a five-year period (2020–2024). Unlike national registries, this institutional dataset includes detailed obstetric and neonatal variables, enabling a nuanced comparison across three clinically meaningful age groups: <15 years, 15–16 years, and 17 years.

The aim of this study was not to compare adolescent pregnancies with adult populations, but to evaluate whether very young maternal age (<15 years) is associated with different obstetric outcomes compared to older adolescents within a hospital-based cohort.

By focusing exclusively on minors and avoiding direct comparisons with adult women, this study seeks to clarify whether younger adolescents experience distinct obstetric risks relative to their older adolescent peers. Given Romania’s unique epidemiological context and the scarcity of detailed clinical research in this field, the findings are intended to support evidence-based clinical decision-making, inform public health policy, and contribute to the development of targeted interventions for adolescent maternal care.

## 2. Materials and Methods

### 2.1. Study Design

All live births and stillbirths among mothers aged <18 years between 1 January 2020 and 31 December 2024 were identified from the institutional electronic birth registry of the Obstetrics and Gynecology Clinic of the Arad County Emergency Clinical Hospital, a secondary maternity hospital with approximately 2300 births per year, providing medical care to both urban and rural populations in western Romania.

Eligible cases included those with complete obstetric documentation, comprising maternal characteristics, intrapartum records, and immediate neonatal data. Cases in which mothers were confirmed with COVID-19 were excluded. This exclusion was applied to avoid potential confounding effects related to SARS-CoV-2 infection, which has been associated with increased risks of preterm birth, cesarean delivery, and maternal complications, potentially biasing the assessment of age-related obstetric outcomes.

The study included only pregnancies resulting in delivery (live birth or stillbirth) recorded in the institutional registry. Pregnancies that ended in early spontaneous abortion or voluntary termination were not included in this dataset, as such cases are not recorded in the obstetric birth registry. Consequently, the effective lower limit of gestational age corresponds to the viability threshold in clinical practice (approximately ≥24 weeks of gestation). Pregnancies ending in early miscarriage or elective termination were not captured in the dataset.

A total of 763 births to adolescents who met the inclusion criteria were recorded during the study period. No cases were excluded due to missing baseline obstetric variables, as information on mode of delivery, parity, and fetal presentation was available for all births. Gestational age (GA) was missing in 45 cases (5.90%), reflecting insufficient prenatal documentation. Comparative analysis revealed no statistically significant differences between cases with and without gestational age history in maternal age distribution, place of residence or parity (all *p* > 0.05), indicating low probability of systematic biases in the two groups.

Mothers were categorized into three predefined age groups to reflect developmental and epidemiological distinctions relevant to adolescent pregnancy research: those younger than 15 years (10–14 years), those aged 15–16 years, and 17-year-old mothers.

A small number of deliveries occurring outside the hospital setting (e.g., ambulance or home births), but documented in the institutional obstetric registry, were included and classified as spontaneous vaginal deliveries, as no instrumental or cesarean procedures were performed.

**Figure 1:**
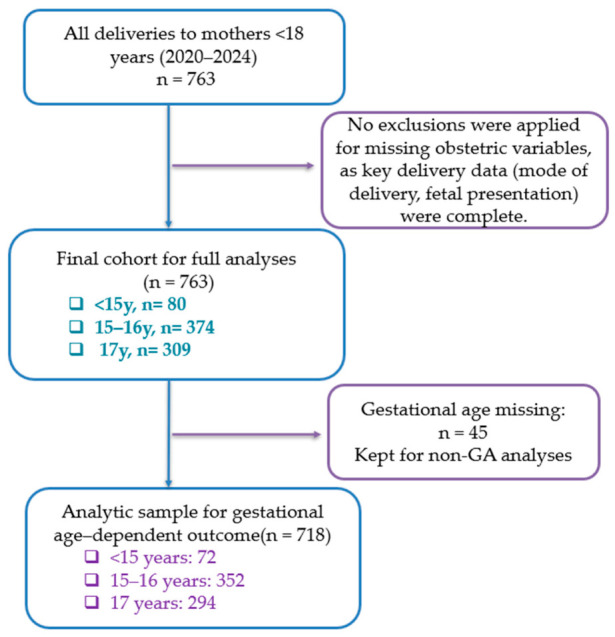
Flowchart of case selection and analytic sample derivation.

A schematic representation of case inclusion and analytic sample derivation is presented in Figure 1.

### 2.2. Data Sources and Collection Procedures

For each case, a standardized electronic dataset was constructed that included demographic, obstetric, intrapartum, and immediate neonatal variables. Maternal age was recorded in completed years. Gestational age was determined using first-trimester ultrasound dating when available, or the date of the last menstrual period otherwise. GA values expressed as ranges (e.g., “36/37 weeks”) were coded as <37 weeks.

Gestational age was analyzed both as a continuous variable and categorically as very preterm (<32 weeks), preterm (32–36^+6^ weeks), and term (≥37 weeks). Because gestational age was not included as a covariate in any regression model—being either an outcome (preterm birth) or an intermediate variable—45 cases with missing GA (5.90%) were retained in all analyses not directly dependent on GA.

Mode of delivery was classified into three categories: spontaneous vaginal delivery, instrumental vaginal delivery (vacuum or forceps), and cesarean section. All vaginal births, with or without oxytocin augmentation or analgesia, were classified as spontaneous unless an instrumental maneuver was documented.

Perineal trauma was classified using the standard four-degree system (grades I–IV). Obstetric anal sphincter injuries (OASIS) were defined as grade III–IV tears. Non-perineal maternal soft-tissue injuries, including cervical, vaginal, paraclitoral, or paraurethral lacerations, were coded separately and summarized using a binary variable (present/absent). Due to their extremely low frequency, perineal tears were analyzed descriptively only and were not included in regression models.

Episiotomy was recorded as a binary variable (yes/no). Additional intrapartum variables included fetal presentation, type of pregnancy (singleton or multiple), membrane status (rupture duration: <12 h, 12–24 h, >24 h), maternal comorbidities, and COVID-19 status at delivery.

For cesarean deliveries, intraoperative maternal complications were classified into predefined clinical categories based on operative reports (Table 1).

### 2.3. Statistical Analysis

All statistical analyses were conducted using JASP (version 0.19.3). Continuous variables were assessed for normality using the Shapiro–Wilk test. As all continuous obstetric and neonatal variables demonstrated non-normal distributions (*p* < 0.05), they are reported as medians and interquartile ranges (IQR). Categorical variables are presented as counts and percentages.

Group comparisons across maternal age categories (<15 years, 15–16 years, and 17 years) were performed using the Kruskal–Wallis test for continuous variables and the χ^2^ test for categorical variables. Effect sizes were reported as ε^2^ for Kruskal–Wallis tests and Cramer’s V for χ^2^ tests, and interpreted according to established thresholds.

Multivariable logistic regression models were constructed to evaluate the independent association between maternal age and two primary obstetric outcomes:

1. Preterm birth (<37 weeks vs. ≥37 weeks)
2. Cesarean delivery (yes/no)

Each outcome was first examined using a minimally adjusted model, followed by a fully adjusted model including clinically relevant covariates: rural residence (yes/no), parity (primiparous vs. multiparous), fetal presentation (non-cephalic vs. cephalic), and duration of membrane rupture (<12 h, 12–24 h, >24 h).

Adjusted odds ratios (aORs) with 95% confidence intervals (CIs) were reported. Model fit was assessed using deviance statistics, Akaike Information Criterion (AIC), Bayesian Information Criterion (BIC), and pseudo-R^2^ indices (McFadden, Nagelkerke, Tjur, Cox & Snell). Multicollinearity was evaluated through standardized coefficients and diagnostic indices.

To explore temporal trends, additional logistic models included year of delivery, maternal age group, and a year × age group interaction term. Predicted probabilities with 95% confidence intervals were derived and visualized using conditional estimates plots.

Statistical significance was set at *p* < 0.05, without adjustment for multiple testing, given the exploratory nature of the analyses.

The study was approved by the Ethics Committee of Arad County Emergency Clinical Hospital (Approval No. 13474/13.04.2022). All data were fully anonymized prior to analysis, and the requirement for individual informed consent was waived due to the retrospective design.

## 3. Results

### 3.1. Maternal Characteristics 

A total of 763 underage mothers were included in the analysis, of whom 309 (40.5%) were aged 17 years, 374 (49.0%) were aged 15–16 years, and 80 (10.5%) were aged <15 years. As expected, maternal age differed significantly across the predefined categories (Kruskal–Wallis χ^2^ = 691.07, *p* < 0.001; ε^2^ = 0.907), reflecting the constructed age grouping rather than a biological gradient.

The distribution of residence was comparable across age groups (χ^2^ = 0.867, *p* = 0.648; Cramer’s V = 0.034). Overall, rural residence predominated, accounting for 60.6% of the cohort, with no meaningful variation according to maternal age (Table 2).

In contrast, parity differed significantly by age group (χ^2^ = 40.75, *p* < 0.001; Cramer’s V = 0.163). Primiparity was most frequent among the youngest mothers, observed in 92.5% of those aged <15 years, compared with 84.2% in the 15–16-year group and 71.2% among 17-year-olds. Conversely, higher-order parities (≥3) were uncommon across the cohort and remained rare in all age categories (≤2.6%) (Table 2).

Missing parity data were minimal (<3% in all groups) and did not differ meaningfully by maternal age.

### 3.2. Obstetric and Intrapartum Characteristics

Overall, obstetric characteristics were comparable across all maternal age groups, with no statistically significant or clinically meaningful differences observed.

**Figure 2:**
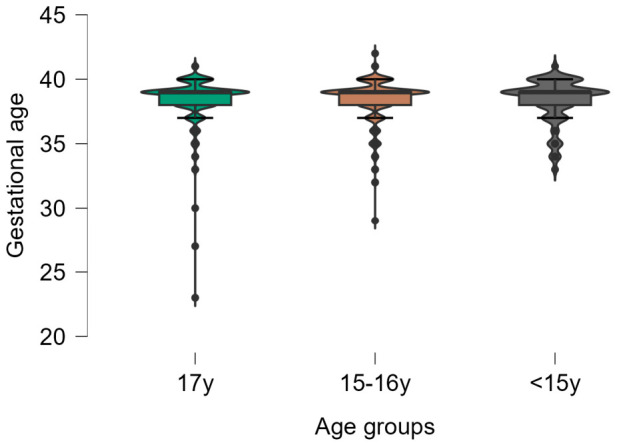
Distribution of gestational age at delivery across maternal age groups.

Gestational age at delivery did not differ significantly across maternal age groups. One-way ANOVA showed no effect of age group on gestational age (F(2715) = 0.02, *p* = 0.998), with a negligible effect size (partial η^2^ < 0.001). Mean gestational age was highly similar among mothers aged <15 years (38.31 ± 1.74 weeks; n = 72), those aged 15–16 years (38.30 ± 1.70 weeks; n = 352), and 17-year-olds (38.29 ± 1.91 weeks; n = 294). A non-parametric sensitivity analysis using the Kruskal–Wallis test confirmed the absence of age-related differences (H = 0.08, *p* = 0.9967; ε^2^ < 0.001). The distribution of gestational age at birth across maternal age categories is illustrated in Figure 2.

Consistent with the continuous analysis, gestational age categories did not differ significantly across maternal age groups (χ^2^ = 3.446, *p* = 0.486; Cramer’s V = 0.049). Very preterm births (<32 weeks) were rare overall (0.55%), while preterm births between 32 and 36 weeks accounted for 11.97% of cases. Many deliveries occurred at term (≥37 weeks; 87.48%), with similar proportions across all age groups.

Fetal presentation showed no significant age-related variation (χ^2^ = 7.423, *p* = 0.492; Cramer’s V = 0.070). Cephalic presentation predominated in all groups (91.25–94.16%), whereas breech and transverse presentations were consistently uncommon (<3%).

Similarly, the duration of membrane rupture did not differ significantly across maternal age categories (χ^2^ = 6.684, *p* = 0.571; Cramer’s V = 0.069). Most patients presented with intact membranes at the onset of labor (85.51–91.78%), and prolonged rupture exceeding 24 h was exceedingly rare (≤0.35%), with no discernible age-related pattern.

The mode of delivery was also comparable across age groups (χ^2^ = 2.280, *p* = 0.684; Cramer’s V = 0.039). Spontaneous vaginal delivery accounted for approximately half of all births (49.2–52.4%), instrumental vaginal delivery remained infrequent (0.65–1.60%), and cesarean section rates ranged from 47.5 to 50.2%, with no significant differences by maternal age (Table 3).

Taken together, these findings indicate that maternal age within the adolescent range was not associated with meaningful variation in gestational age, intrapartum characteristics, or mode of delivery, with all observed effect sizes falling within the negligible range.

### 3.3. Intrapartum and Maternal Morbidity Outcomes

#### 3.3.1. Vaginal Birth Outcomes

Among vaginal births, the frequency of episiotomy was high across all maternal age groups and did not differ significantly by age. Episiotomy was performed in 76.2% of births among 17-year-old mothers, 77.1% among those aged 15–16 years, and 85.4% among mothers younger than 15 years (χ^2^ = 1.63, *p* = 0.442).

Maternal non-perineal soft-tissue injuries (binary outcome) occurred at comparable rates across age groups. CPMM was recorded in 29.1% of vaginal births among 17-year-olds, 29.5% among mothers aged 15–16 years, and 30.8% among those younger than 15 years, with no statistically significant association between maternal age and soft-tissue injury (χ^2^ = 0.04, *p* = 0.978; Cramer’s V = 0.01), indicating a negligible effect size (Table 4). Cervical and vaginal lacerations accounted for the majority of recorded soft-tissue injuries, while paraurethral and paraclitoridian lesions were uncommon. Perineal tears were rare in the cohort, with only two cases identified overall (one grade II tear among 17-year-olds and one grade I–II tear among mothers aged 15–16 years).

#### 3.3.2. Cesarean Delivery–Related Maternal Morbidity

When analyzed by detailed complication categories, no significant differences were observed (χ^2^ = 15.90, df = 10, *p* = 0.103). When grouped as a binary outcome (any complication vs. none), the association remained non-significant (χ^2^ = 3.79, *p* = 0.150). Individual complication types, including hemorrhagic events, uterine dehiscence, surgical adhesions, adnexal pathology, and hysterectomy, were rare and occurred sporadically across age groups.

For analytic purposes, cesarean-related maternal morbidity was also examined as a binary outcome (any maternal surgical complication vs. none), which similarly showed no significant association with maternal age (χ^2^ = 3.79, *p* = 0.150) (Table 5).

### 3.4. Multivariable Regression Analysis

Two multivariable logistic regression models were constructed to evaluate whether maternal age independently predicted key obstetric outcomes in adolescent pregnancies. The outcomes examined were preterm birth (<37 weeks) and cesarean delivery (yes/no).

#### 3.4.1. Preterm Birth

In the fully adjusted model for preterm birth, maternal age was not independently associated with delivery before 37 weeks (all *p* > 0.05). Compared with mothers aged 17 years, the adjusted odds ratio (aOR) was 0.80 (95% CI 0.49–1.31; *p* = 0.375) for those aged 15–16 years and 0.80 (95% CI 0.36–1.77; *p* = 0.583) for mothers aged <15 years. None of the included covariates—parity, residence, or duration of membrane rupture—reached statistical significance. Overall model performance was limited (Nagelkerke R^2^ = 0.016; McFadden R^2^ = 0.011), and the model did not significantly improve fit compared with the intercept-only model (Δχ^2^ *p* = 0.767) (Table 6).

#### 3.4.2. Cesarean Delivery

Maternal age was likewise not associated with cesarean delivery. Relative to 17-year-olds, the adjusted odds ratios were 0.80 (95% CI 0.58–1.10) for mothers aged 15–16 years and 0.83 (95% CI 0.49–1.42) for those aged <15 years. Cesarean delivery was strongly associated with non-cephalic fetal presentation (aOR 7.20, 95% CI 2.11–24.61; *p* = 0.002). In contrast, shorter duration of membrane rupture (<12 h and ≥12 h) was associated with reduced odds of cesarean delivery. Parity and rural residence did not independently predict surgical birth (Table 7). The model demonstrated modest explanatory capacity (Nagelkerke R^2^ = 0.053).

#### 3.4.3. Temporal Trends in Obstetric Outcomes (2020–2024)

To explore potential changes in obstetric outcomes over time, additional logistic regression models including year of delivery, maternal age group, and their interaction were constructed. No significant year × age group interaction was identified for preterm birth, cesarean delivery, or major intrapartum complications (all interaction *p*-values > 0.10).

**Figure 3:**
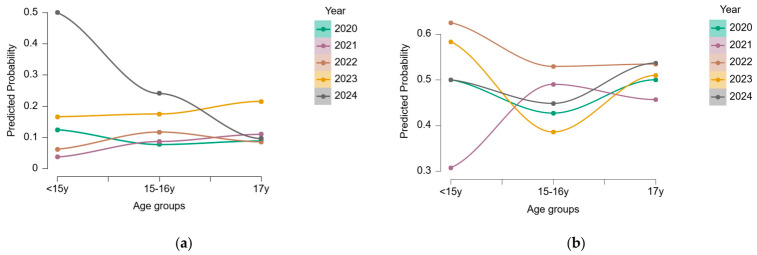
Annual predicted probability of (**a**) preterm birth and (**b**) cesarean delivery among adolescent mothers, stratified by maternal age group, 2020–2024.

Predicted probability plots demonstrated stable outcome patterns across the study period, with no consistent or monotonic trends by maternal age (Figure 3). These findings suggest temporal stability of obstetric outcomes among adolescent mothers despite the COVID-19 pandemic period.

## 4. Discussion

This retrospective analysis of 763 births among underage mothers provides new insights into obstetric outcomes of adolescent pregnancies managed in a secondary maternity setting in Romania. The main findings indicate that maternal chronological age—even among very young adolescents (<15 years)—was not independently associated with preterm birth or cesarean delivery. Instead, obstetric and intrapartum factors such as fetal presentation, membrane rupture status, and parity were the principal determinants of outcomes. These results support the interpretation that, when adolescent pregnancies are managed in adequately equipped medical institutions with standardized intrapartum surveillance, biological age alone may exert a limited influence on obstetric morbidity.

The results of this study align with evidence from Romanian cohorts reporting comparable obstetric outcomes among early and late adolescents when continuity of care is ensured. In Southeastern Romania, Brezeanu et al. observed no significant age-related differences in preterm birth or cesarean section after adjustment for anemia and attendance at prenatal care [17]. Similarly, Matasariu et al. found that perinatal outcomes among younger adolescents (13–16 years) and older adolescents (17–19 years) were statistically equivalent after controlling for confounders such as parity and antenatal follow-up [16]. A multicentric study from northeastern Romania further demonstrated that social determinants—rural residence, education, and timing of prenatal visits—were stronger predictors of adverse outcomes than age per se [18].

Comparable trends have been described across Central and Eastern Europe. In Poland, Manasar-Dyrbuś et al. reported that adolescent age did not independently predict preterm birth or operative delivery after accounting for clinical conditions [19]. Taken together, these findings suggest that the traditionally perceived obstetric vulnerability of adolescent mothers may be attenuated in healthcare contexts where antenatal surveillance and institutional delivery are accessible [18,19,20,21].

At a global level, the literature remains more heterogeneous. The WHO multicountry study by Ganchimeg et al., which included births from 29 countries, reported that very young adolescents had increased risks of preterm delivery and low birth weight, particularly in settings where access to prenatal care was limited [20]. More recent regional evidence also indicates that health system factors and antenatal care coverage modify the magnitude of risk associated with adolescent pregnancy. A meta-analysis from the Eastern Mediterranean region reported that adverse outcomes are common but vary substantially across settings, highlighting the importance of contextual determinants and healthcare quality [21]. Mothers who had babies at a young age (< 15 years) do not have worse obstetric outcomes than older adolescent mothers within this adolescent population. These findings should not be viewed as indicating a lack of risk to the general obstetric population.

The absence of significant associations between age and obstetric complications in this cohort can be interpreted in light of both biological and sociomedical mechanisms. Several studies suggest that gynecologic age (the interval between menarche and conception), rather than chronological age, may better capture biological immaturity and its relationship to the risk of preterm birth in very young adolescents [22,23]. Unfortunately, age at menarche and gynecological age were not available in the current dataset, preventing direct assessment of this dimension of biological risk. Future research should take into account the age at first menstruation and calculate gynecological age as a more accurate indicator of biological maturity, which could enable improved risk stratification and a better understanding of the relationship between developmental stage and obstetric outcomes among adolescents [22,23].

Social and behavioral determinants remain critical contributors to adolescent pregnancy outcomes. Delayed prenatal presentation, limited prenatal visit frequency, low educational level, and nutritional vulnerability have repeatedly been associated with prematurity and poorer neonatal outcomes among adolescent mothers [5]. These vulnerabilities are further exacerbated by systemic limitations within health systems, particularly in contexts where access to timely and comprehensive maternal care is uneven [24].

In Romania and neighboring countries, where adolescent fertility remains among the highest in Europe, structured antenatal care and institutional deliveries may buffer risks that are otherwise amplified in low-resource environments [17,25]. Despite the existence of a mixed healthcare model, with both public and private providers, persistent challenges such as insufficient funding, shortages of medical personnel, and inefficiencies in service delivery continue to undermine equitable access to care [24,26]. These deficiencies are particularly pronounced in rural and peripheral regions, where medical infrastructure is weaker and labor distribution is suboptimal. As a result, vulnerable populations, including adolescent mothers, are disproportionately disadvantaged. This interaction between social and clinical factors underscores the fact that negative pregnancy outcomes among adolescents can often be prevented when comprehensive maternal care is provided [20,27,28].

The cesarean section rate observed in this study (approximately 50%) reflects both local clinical practice and broader national trends. Similarly high rates have been reported in Romanian tertiary centers [18,29] and in other middle-income European contexts [21]. Notably, maternal age was not an independent determinant of cesarean birth in our models; instead, non-cephalic presentation and intrapartum characteristics, including membrane rupture status, were the strongest predictors, consistent with prior Romanian adolescent cohorts [28].

Global datasets likewise indicate that cesarean delivery decisions are largely obstetric rather than age-driven when standardized intrapartum protocols are applied. From a clinical perspective, these findings support a therapeutic approach in which adolescent mothers are not considered a high-risk group solely on the basis of age, but are assessed according to standard obstetric criteria. This underscores the importance of avoiding age-based clinical biases and supports adherence to evidence-based intrapartum protocols when determining the mode of delivery. In practice, this can help reduce the number of unnecessary cesarean sections and promote the appropriate use of surgical delivery based on fetal presentation, labor progress, and maternal-fetal status. Ensuring equitable access to skilled obstetric care and continuous intrapartum monitoring remains essential for achieving optimal outcomes in this population [20,30]. A secondary analysis of the WHO Global and Multicountry Surveys examining singleton preterm births further supports the concept that clinical indications and obstetric context are central to delivery mode decision-making [30]. In line with this evidence, our findings argue against cesarean indication solely based on maternal youth, provided that adequate intrapartum monitoring, surgical expertise, and neonatal support are ensured. The relatively high cesarean section rate may also reflect institutional practices, medico-legal considerations, and clinician preference in managing adolescent pregnancies, particularly in contexts where continuous fetal monitoring and risk aversion influence decision-making.

No significant age-related differences were observed in episiotomy rates or non-perineal maternal soft-tissue injuries in our study, consistent with multicentric Romanian data assessing obstetrical soft tissue trauma in adolescent populations [25]. Severe perineal trauma was rare in the present cohort and was therefore reported descriptively only.

The relatively high rate of cesarean deliveries observed in this study (approximately 50%) reflects both local clinical practice and broader national trends. Similarly high rates have been reported in tertiary care centers in Romania [18,29] and in other middle-income European countries [21]. It is important to note that maternal age was not an independent predictor of cesarean delivery in our models. Instead, non-cephalic presentation and intrapartum characteristics, including the status of membrane rupture, were the main determinants, consistent with previous findings in adolescent populations [28]. These results support the interpretation that mode of delivery among adolescents is largely influenced by obstetric indications and health system factors, rather than chronological age itself.

The low prevalence of instrumental vaginal deliveries may have contributed to the overall reduced rates of genital tract injury. Evidence suggests that maternal soft-tissue trauma is more strongly associated with fetal size, duration, and mechanics of the second stage of labor, and operator technique than with maternal age per se [25]. Thus, the present findings support the view that the quality of intrapartum management—particularly controlled delivery of the fetal head—is a decisive factor for perineal and soft-tissue outcomes in adolescents.

Across the present study period (2020–2024), no significant temporal variations were detected in preterm birth, cesarean delivery, or intrapartum maternal complications, despite overlap with the COVID-19 pandemic. This temporal stability mirrors Romanian findings reported over comparable periods [31] and has also been described in Polish cohort analyses [19].

The fact that such a high number of women are receiving episiotomies (76–85%) may indicate that this is likely due to the repetitive nature of clinicians at each of these institutions performing most of their episiotomies on an adolescent population, in fear of perineal trauma, and/or based on operator modality preference. Furthermore, this practice of routinely performing episiotomies could play a significant role in maternal morbidity and, therefore, requires more examination in the current context of international guidelines, which recommend a more restrictive approach to the use of episiotomies.

The strengths of this study include its cohort size, inclusion of the full adolescent age spectrum, and standardized extraction of detailed intrapartum variables. However, the retrospective design and missing gestational age data in a small proportion of cases (5.90%) represent important limitations. Moreover, the dataset lacked information on nutritional status, educational attainment, and timing of antenatal care initiation, factors repeatedly linked to prematurity risk and adverse neonatal outcomes in adolescent pregnancies. A synthesis of adolescent pregnancy outcomes further emphasizes the role of prenatal care engagement and contextual disadvantage in shaping maternal and neonatal risk.

Another interesting finding was the association between a shorter duration of membrane rupture and a lower likelihood of cesarean delivery. This may reflect differences in labor progression and clinical management. A shorter duration of membrane rupture is generally associated with earlier stages of labor and more favorable obstetric conditions, reducing the likelihood of an operative delivery. In contrast, prolonged rupture of membranes has been associated with increased risks of intraamniotic infection, labor dystocia, and fetal distress, all of which are recognized indications for cesarean section [32,33,34]. Furthermore, prolonged rupture of membranes may lead to earlier clinical intervention due to concerns about maternal and neonatal infection, thereby increasing cesarean section rates [34]. These findings suggest that the duration of membrane rupture may act as an important intrapartum factor influencing mode of delivery, independent of maternal age.

Insufficient data were provided for the analysis of antenatal complications (i.e., hypertensive disorders) such as fetal growth restriction, maternal comorbid conditions (e.g., diabetes), behavioral factors (i.e., tobacco smoking, alcohol consumption), body mass index (i.e., obesity) and the adequacy of antenatal care provided. All of these factors have been shown to affect obstetric outcomes, so their omission creates another major limitation for this analysis.

Clinically, these findings suggest that comprehensive prenatal follow-up and skilled obstetric management within a hospital-based setting may mitigate a substantial proportion of the potential disadvantage associated with very young maternal age. From a public health perspective, however, Romania continues to report persistently elevated adolescent fertility rates, driven by complex social determinants [35]. Prevention strategies should therefore prioritize sexual health education, community outreach, and access to contraception, while maintaining robust intrapartum and perinatal care systems. Evidence from Central and Eastern Europe and UNPF regional analyses supports the effectiveness of integrated healthcare and education-based interventions in reducing adolescent birth rates [36].

The study period overlapped with the COVID-19 pandemic, which has been shown to influence maternal and perinatal outcomes, including through changes in healthcare access and infection-related risks. However, no significant temporal variations were observed in our cohort. Similar findings regarding SARS-CoV-2 exposure in pregnant populations have been reported in previous studies [37,38]. However, no significant changes over time were observed in our cohort. Similar findings have been reported in other studies evaluating obstetric outcomes during the pandemic, suggesting relative stability in key maternal outcomes in settings where access to institutional medical care was maintained [31,37,38,39].

Compared to international benchmarks, the elevated rate may reflect broader national trends in Romania, such as the medico-legal aspects associated with these indicators and the degree of patient preference and/or demand for these services.

There are many advantages to this analysis: it involves many children; covers a broad spectrum of adolescents; and provides very detailed, complete sets of institutionalized records that allow for individualized results by age group. This would allow for a far more detailed examination of the factors that influence pregnancy outcomes in children, unlike national studies, where details about the circumstances surrounding the pregnancy are not always available.

There are several limitations to be noted. These include: the retrospective design of this study; the absence of a control group for adult mothers; and the limited availability of necessary clinical/social variables (antenatal complications, maternal behaviors, nutritional status, and neonatal outcomes). All these factors may affect the ability to interpret the study’s findings and, as such, limit the study’s generalizability. In addition, the single-center design may limit the generalizability of the findings to other healthcare settings with different patient populations, resource availability, and clinical practice patterns. The absence of key antenatal and behavioral variables, including maternal comorbidities, use of prenatal care, and lifestyle factors, represents a potential source of residual confounding. The lack of standardized data on postpartum outcomes, including length of hospital stay, readmissions, and postpartum maternal morbidity, limits the ability to assess the full spectrum of maternal risks. Furthermore, neonatal outcomes such as birth weight, Apgar score, and neonatal intensive care unit admission were not available.

In summary, this study provides evidence that, within an institutional obstetric care setting, maternal chronological age below 18 years does not independently predict adverse obstetric outcomes. Instead, obstetric factors and broader social determinants of health appear to be more important drivers of maternal and neonatal wellbeing. Future strategies should therefore balance clinical optimization with upstream prevention, ensuring that adolescent pregnancy is addressed not only as a medical event but also as a multidimensional public health issue.

There was no information available on the three (3) neonatal outcomes that could have assisted this analysis (birth weight, Apgar scores, and NICU admission). Therefore, researchers were unable to fully assess the extent of the perinatal risk spectrum.

## 5. Conclusions

In this hospital-based cohort of underage mothers, no significant differences in obstetric outcomes were observed between adolescent age groups after adjustment for relevant clinical factors. Preterm birth, mode of delivery, and intrapartum maternal morbidity were comparable across age categories, including among very young adolescents (<15 years).

Obstetric outcomes appeared to be primarily influenced by intrapartum characteristics, particularly fetal presentation and membrane rupture status, rather than maternal chronological age within the adolescent population. Outcome patterns remained stable throughout the 2020–2024 study period, including during the COVID-19 pandemic, indicating consistency of obstetric care and management within the study institution. Importantly, these findings are limited to comparisons within adolescent subgroups and do not allow inference regarding risk relative to adult populations.

Adolescent pregnancy is an important concern both from a social and public health perspective, influenced by complex socioeconomic determinants, including limited access to education, healthcare, and reproductive services.

Future studies should integrate indicators of biological maturity and social vulnerability, including age at menarche, nutritional status, and timing of antenatal care initiation, to better elucidate risk pathways in adolescent pregnancy.

## Figures and Tables

**Table 1:** Categories of intraoperative maternal complications during cesarean delivery.

Category	Included Events
No maternal complication	Uneventful surgery
Hemorrhage/hematoma/abruption	Hemorrhage, retroplacental hematoma
Uterine rupture/dehiscence	Complete or partial
Adhesions/difficult surgery	Peritoneal adhesions
Visceral injury	Bladder or other organ injury
Adnexal pathology	Chistectomy, adnexal tumor removal
Hysterectomy	Any indication

**Table 2:** Maternal characteristics by age group.

Variable	<15 Years (n = 80)	15–16 Years (n = 374)	17 Years (n = 309)	Statistics	*p*-Value	Effect Size
Maternal age (years), mean ± SD	13.74 ± 0.50	15.62 ± 0.48	17.00 ± 0.00	χ^2^ = 691.07 ^a^	<0.001	ε^2^ = 0.907
Residence						
Rural	45 (56.25%)	231 (61.76%)	186 (60.19%)	χ^2^ = 0.867	0.648	*V* = 0.034
Urban	35 (43.75%)	143 (38.24%)	123 (39.81%)			
Parity						
Para 1	74 (92.50%)	315 (84.22%)	220 (71.17%)	χ^2^ = 40.75	<0.001	*V* = 0.163
Para 2	4 (5.00%)	51 (13.64%)	69 (22.33%)			
Para 3	0 (0.00%)	3 (0.80%)	17 (5.50%)			
Para ≥ 4	0 (0.00%)	2 (0.53%)	0 (0.00%)			
Missing value	2 (2.50%)	3 (0.80%)	3 (0.97%)			

^a^ Kruskal–Wallis test. *p*-values reflect global group comparisons across maternal age categories. Effect sizes are reported as epsilon squared (ε^2^) for continuous variables and Cramer’s V (V) for categorical variables.

**Table 3:** Obstetric and intrapartum characteristics by maternal age group.

Variable	<15 Years (n = 80)	15–16 Years (n = 374)	17 Years (n = 309)	Statistics	*p*	Effect Size
Gestational age category						
<32 weeks	0 (0.00%)	1 (0.28%)	3 (1.02%)	χ^2^ = 5.731	0.251	*V* = 0.062
32–36^+6^ weeks	10 (13.89%)	46 (13.07%)	30 (10.20%)			
≥37 weeks	62 (86.11%)	305 (86.65%)	261 (88.78%)			
Missing value	8 (10.00%)	22 (5.88%)	15(4.85%)	-	-	-
Fetal presentation						
Cephalic	73 (91.25%)	351 (93.85%)	290 (94.16%)	χ^2^ = 7.423	0.492	*V* = 0.070
Breech	2 (2.50%)	13 (3.74%)	6 (1.95%)			
Transverse	1 (1.25%)	0 (0.00%)	2 (0.65%)			
Missing/other	4 (5.00%)	10 (2.67%)	11 (3.60%)	-	-	-
Duration of membrane rupture						
Intact	67 (91.78%)	309 (89.31%)	242 (85.51%)	χ^2^ = 6.684	0.571	*V* = 0.069
<12 h	1 (1.37%)	3 (0.87%)	7 (2.47%)			
13–24 h	0 (0.00%)	11 (3.18%)	12 (4.24%)			
>24 h	0 (0.00%)	1 (0.29%)	1 (0.35%)			
Missing value	5 (6.85%)	22 (6.35%)	21 (7.43%)	-	-	-
Delivery mode						
Spontaneous vaginal	41 (51.25%)	196 (52.41%)	152 (49.19%)	χ^2^ = 2.280	0.684	*V* = 0.039
Instrumental vaginal	1 (1.25%)	6 (1.60%)	2 (0.65%)			
Cesarean section	38 (47.50%)	172 (45.99%)	155 (50.20%)			

Data are presented as absolute numbers (n) and percentages (%), calculated based on valid cases only. Missing data were excluded from inferential analyses. *p*-values reflect global group comparisons across maternal age categories. Effect sizes are reported as Cramer’s V (V) for categorical variables.

**Table 4:** Vaginal birth outcomes by maternal age group.

Variable	<15 Years (n = 41)	15–16 Years (n = 196)	17 Years (n = 152)	Statistics	*p*-Value	Effect Size
Episiotomy						
Yes	35 (85.4%)	148 (77.1%)	115 (76.2%)	χ^2^ = 1.63	0.442	*V* = 0.07
No	6 (14.6%)	44 (22.9%)	36 (23.8%)			
Missing	0 (0.00%)	4(2.04%)	1(0.65%)	-	-	-
Maternal soft-tissue injury						
Yes	12 (30.8%)	57 (29.5%)	43 (29.1%)	χ^2^ = 0.04	0.978	*V* = 0.01
No	27 (69.2%)	136 (70.5%)	105 (70.9%)			
Missing value	2 (4.9%)	3 (1.5%)	4 (2.6%)	-	-	-

Percentages and statistical tests are based on valid cases only. Missing data were excluded from inferential analyses. *p*-values reflect global group comparisons across maternal age categories. Effect sizes are reported as Cramer’s V (V) for categorical variables.

**Table 5:** Cesarean delivery outcomes by maternal age group.

Maternal Complication	<15 Years (n = 38)	15–16 Years (n = 172)	17 Years (n = 155)	Statistics	*p*-Value	Effect Size
No maternal complication	33 (86.8%)	165 (96.5%)	145 (94.2%)	χ^2^ = 3.79	0.150	*V* = 0.15
Any maternal surgical complication	5 (13.2%)	6 (3.5%)	9 (5.8%)			
Missing value	0 (0.00%)	1(0.58%)	1(0.65%)	-	-	-

Percentages and statistical tests are based on valid cases only. Missing data were excluded from inferential analyses. *p*-values reflect global group comparisons across maternal age categories. Effect sizes are reported as Cramer’s V (V) for categorical variables.

**Table 6:** Multivariable logistic regression models for preterm birth.

Predictor	aOR	95% CI	*p*
Age 15–16 vs. 17	0.80	0.49–1.31	0.375
Age < 15 vs. 17	0.80	0.36–1.77	0.583
Parity ≥ 2 vs. 1	1.00	0.56–1.77	0.987
Urban vs. rural	0.92	0.59–1.46	0.735
Non-cephalic presentation	0.47	0.16–1.39	0.170
Membrane rupture ≥ 12 h	3.05	0.34–27.27	0.318
Membrane rupture < 12 h	1.20	0.20–7.27	0.846

**Table 7:** Multivariable logistic regression models for cesarean delivery.

Predictor	OR	95% CI	*p*-Value
Age 15–16 vs. 17	0.80	0.58–1.10	0.171
Age < 15 vs. 17	0.83	0.49–1.42	0.502
Primipara	1.08	0.74–1.59	0.690
Rural residence	1.00	0.73–1.36	0.991
Non-cephalic	7.20	2.11–24.61	0.002
MR < 12 h	0.46	0.26–0.82	0.009
MR ≥ 12 h	0.37	0.15–0.91	0.031

## Data Availability

The datasets are not publicly available; however, Florin Mihai Sandor may provide de-identified data upon request.

## Abbreviations

The following abbreviations are used in this manuscript:

GA	Gestational Age
INS	Institutul Național de Statistică (National Institute of Statistics, Romania)
IQR	Interquartile Range
MR	Membrane Rupture
OASIS	Obstetric Anal Sphincter Injuries (grade III–IV perineal tears)
UNFPA	United Nations Population Fund
WHO	World Health Organization
